# Efficiently Combining Water Reuse and Desalination through Forward Osmosis—Reverse Osmosis (FO-RO) Hybrids: A Critical Review

**DOI:** 10.3390/membranes6030037

**Published:** 2016-07-01

**Authors:** Gaetan Blandin, Arne R.D. Verliefde, Joaquim Comas, Ignasi Rodriguez-Roda, Pierre Le-Clech

**Affiliations:** 1 LEQUIA, Institute of the environment, University of Girona, Campus Montilivi, Girona 17003, Spain; joaquim.comas@udg.edu (J.C.); irodriguezroda@icra.cat (I.R.-R.); 2 Department of Applied Analytical and Physical Chemistry, Ghent University, Faculty of Bioscience Engineering, Particle and Interfacial Technology Group (PaInT), Gent 9000, Belgium; arne.verliefde@ugent.be; 3 UNESCO Centre for Membrane Science and Technology, School of Chemical Engineering, The University of New South Wales, Sydney NSW2052, Australia; p.le-clech@unsw.edu.au; 4 ICRA, Catalan Institute for Water Research, Parc scientific and technologic of the university of Girona, Girona 17003, Spain

**Keywords:** potable water reuse, seawater desalination, pressure assisted osmosis, module, fouling, trace organic contaminants

## Abstract

Forward osmosis (FO) is a promising membrane technology to combine seawater desalination and water reuse. More specifically, in a FO-reverse osmosis (RO) hybrid process, high quality water recovered from the wastewater stream is used to dilute seawater before RO treatment. As such, lower desalination energy needs and/or water augmentation can be obtained while delivering safe water for direct potable reuse thanks to the double dense membrane barrier protection. Typically, FO-RO hybrid can be a credible alternative to new desalination facilities or to implementation of stand-alone water reuse schemes. However, apart from the societal (public perception of water reuse for potable application) and water management challenges (proximity of wastewater and desalination plants), FO-RO hybrid has to overcome technical limitation such as low FO permeation flux to become economically attractive. Recent developments (i.e., improved FO membranes, use of pressure assisted osmosis, PAO) demonstrated significant improvement in water flux. However, flux improvement is associated with drawbacks, such as increased fouling behaviour, lower rejection of trace organic compounds (TrOCs) in PAO operation, and limitation in FO membrane mechanical resistance, which need to be better considered. To support successful implementation of FO-RO hybrid in the industry, further work is required regarding up-scaling to apprehend full-scale challenges in term of mass transfer limitation, pressure drop, fouling and cleaning strategies on a module scale. In addition, refined economics assessment is expected to integrate fouling and other maintenance costs/savings of the FO/PAO-RO hybrid systems, as well as cost savings from any treatment step avoided in the water recycling.

## 1. Introduction

### 1.1. Need for Alternative Water Resources and Management

With the world population ever increasing, water scarcity and resource depletion have become pressing problems. In 2015, 660 million people in the world were lacking access to clean and safe drinking water [1]. With fresh water resources becoming increasingly limited, depleted or contaminated, diversification of water sources is seen as a key evolution in water management, especially in regions facing water scarcity or drought [2]. Current water management strategies are increasingly focusing on the importance of water reuse and seawater desalination as alternative water sources to solve issues of water shortage. In 2018, desalinated water production (from brackish and seawater) is forecasted to exceed 36 billion m^3^ worldwide [3,4] whereby seawater desalination represents more than 60% of the installed capacity. Water reuse is also increasingly considered. As most of the wastewater withdrawn for human activity is currently still being returned to the environment rather than being treated for reuse, reuse of water holds a great potential as alternative water source.

Seawater desalination and water reuse schemes have already been implemented worldwide, but their broader development remains limited due to both public perception and overall treatment costs/energy usage. In fact, it has been shown that public acceptance of alternative water scenarios is mainly driven by the lack of conventional water sources, i.e., only if there is real water shortage, acceptance is increased [5]. It is clear that better education of the public on alternative water sources, and increased awareness of water scarcity are of utmost importance [6,7]. However, broader implementation of alternative water schemes also requires technical progress to ensure safe drinking water (high and constant level of pollutant rejection) at lower treatment costs [8].

### 1.2. State of the Art of Desalination and Water Reuse Schemes

In terms of seawater desalination, reverse osmosis (RO) is the fastest growing technique, and it has taken the leading position in the market, as a result of its lower water production costs compared to thermal desalination processes such as multi-stage flash (MSF) and multi effect distillation (MED) [9]. The market growth of RO seawater desalination (SWRO) has been mainly driven by important progress in reduction of energy demand, from 10 kWh·m^−3^ in 1980 to less than 4 kWh·m^−3^ nowadays. Modern large-scale RO desalination plants go down to 2.2 kWh·m^−3^ specific energetic consumption, and some pilot plants are even operating at 1.8 kWh·m^−3^ [4,10]. Further improvements are theoretically still possible by optimising RO operation (i.e., process control, RO configurations, recovery and fouling mitigation [11,12,13]) down to the thermodynamical limit of 1.06 kWh·m^−3^ (for 50% feed water recovery), but it is clear that RO is really approaching the limit [4]. However, energy costs related to the pre- and post-treatment processes are also an important aspect of the overall desalination expenses [3,11]. For example, energy consumption of some pre-treatment options is higher than 1 kWh·m^−3^. This of course drives the search for either higher water recovery in the RO, or lower energy demand of the whole treatment scheme, or a combination of both. The current cost of seawater desalination is evaluated in average around 0.76 US $·m^−3^, but typically falls within a wide range of 0.5–2 US $·m^−3^—depending mainly on local energy cost [4]. As such desalination remains quite costly, limiting its broader usage. Operational costs (OPEX—include energy and all other costs associated to maintenance, labour and the use of chemicals) account for two third of the total desalination costs for full-scale plants, while the last third of the costs is related to capital cost (CAPEX). In the OPEX, energy accounts for about half of the cost.

As an alternative to RO seawater desalination, water reuse through advanced wastewater treatment plants can also technically provide water of drinking water quality, but the main challenge in (potable) water reuse so far has been to set best practices, policies and high control standards to increase public acceptance [14,15]. Moreover, the removal of (organic) micropollutants (also called trace organic contaminants, TrOCs) that are not fully removed by conventional (biological) wastewater treatment plants [16,17] requires specific attention. As such, planned indirect potable reuse (IPR), which consists of blending an extensively treated wastewater with another source of fresh water, for example through recharging the treated wastewater into a subsurface ground water or into an above-ground surface water reservoir before drinking water treatment, is currently the most used in water reuse schemes. In this case, the reservoir acts as environmental buffer and the drinking water purification step provides an additional barrier to potential pollution. Planned IPR schemes are already in use in few places of the world [18] such as Singapore, Belgium, California and Australia. However, implementation of these schemes can require extensive pumping costs related to transport of the treated effluent back to upstream reservoirs, which is affecting their economic viability [19]. Alternatively, direct potable reuse (DPR) implies the injection of extensively treated wastewater within the local drinking water supply. Such scheme requires even more stricter control than IPR of wastewater treatment but may avoid extensive piping and pumping costs [19].

**Figure 1:**
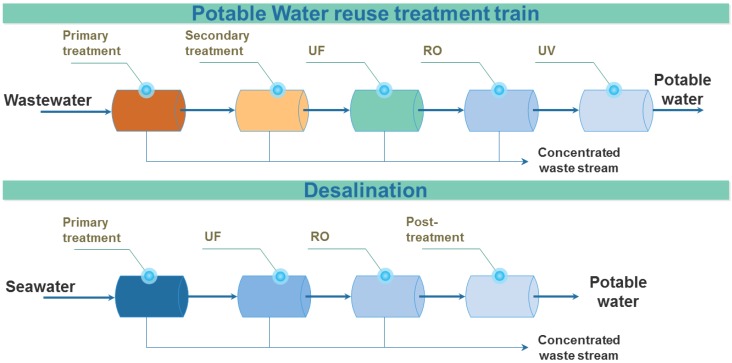
Examples of typical potable water reuse and desalination treatment trains, based on case studies in [18,26].

For both IPR and DPR, to assure drinking water quality and to avoid health risk of such scheme especially with regards to organic contaminants, pathogens and TrOCs, the multiple barrier approach has been developed [20,21]. Specific treatment towards TrOCs removal or degradation were assessed and implemented: dense membrane technologies such as nanofiltration (NF) or RO [22,23], advanced oxidation or adsorption on active carbon proved to be efficient treatments [22,24,25]. Water reuse treatment consists of pursuing the purification of a secondary treated wastewater through an advanced wastewater treatment plant. Typically, such plant consists in passing through two sets of membrane processes (for example ultrafiltration (UF) and RO) and a disinfection step (ultraviolet, ozonation) as described in [18,26] and Figure 1. As a result of such an extensive treatment train, direct potable water reuse remains as costly as desalination, with main case studies and practical examples providing numbers in the range of 0.69–1.23 $·m^−3^ of water produced [18].

In practice, desalinated seawater remains the main alternative source for drinking water, while water reuse is mainly dedicated to irrigation or industrial purposes and as such, both streams are very distinct. Seawater desalination is therefore the first option for safe drinking water production but its energy consumption remains the main obstacle. Ultimately, both seawater desalination and water reuse schemes require further improvement and more attractive economics to allow for broader development.

### 1.3. Opportunities and Challenges of Combining Desalination and Water Reuse Schemes

**Figure 2:**
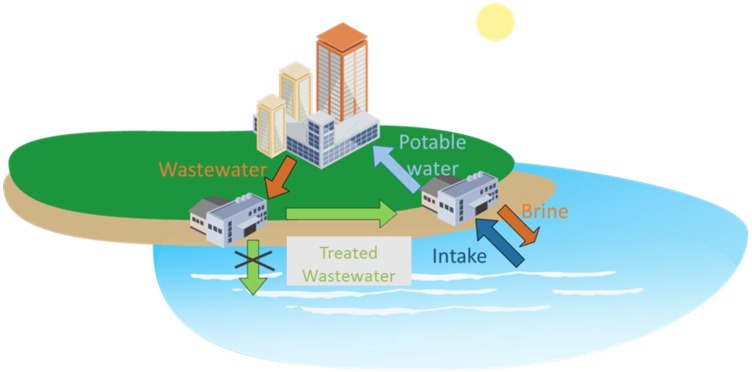
Potential combination of wastewater reuse and seawater desalination to support potable water needs.

Forty percent of the world’s population lives in urban coastal areas, which are typically faced with the joint presence of multiple water sources of different qualities and salinity levels (e.g., river water, wastewater, seawater…). In several densely populated (dry) coastal regions, water is not reused, but drinking water is produced from seawater desalination. In these cases, typically wastewater treatment plant effluents and seawater intake points are in a relatively close geographic area (as illustrated in Figure 2). In other examples, water reuse and seawater desalination are both implemented, such as in California or several regions in Australia. In Singapore, since the implementation of the NEWater program, both water reuse and desalination participate to the overall potable water supply but through distinct water purification scheme [27]. So far, water reuse and desalination have always been considered as separate and independent streams to solving water shortage.

Implementation of a single scheme combining both concepts requires some prerequisites such as proximity of both streams, and ideally the location of water reuse and desalination facilities in one place, which require long term planning in water management, along with technical and economic justification. However, combining a desalination facility with another plant, also called co-sitting scheme, have already been proposed by implementing desalination close to a power plant to lower water intake costs, optimise energy efficiency and eventually combine water streams [28]. This concept has been extended to hybrid systems, such as combined RO-multi-stage flash (MSF) distillation systems [29], or membrane distillation (MD) [30]. As already demonstrated in other co-sitting plants, integrating wastewater treatment and desalination in one plant can also result in potential economic benefits [28]. As such, combining water reuse and desalination schemes with the FO-RO hybrid process (Figure 1) could present major advantages in water management, as combining these schemes could synergistically lower water intake costs and could optimise energy efficiency of water treatment. The technical and economic feasibility to combine water reuse and desalination by this FO-RO hybrid will be discussed in detail hereafter.

## 2. Combining Desalination and Water Reuse through FO-RO Hybrids

### 2.1. The Emergence of FO

In the last 10 years, a growing interest has been observed in osmotically driven processes, also called engineered osmosis. The work of Loeb in the 1970s remained relatively unexplored until new semi-permeable membranes, tailored-made for osmosis applications were developed in the early 2000’s, and commercialised by Hydration Technologies Innovations (HTI) [31,32,33]. The two main osmotically-driven processes that were considered until recently are defined as forward osmosis (FO) and pressure retarded osmosis (PRO). In such systems, the solute concentration gradient (also called osmotic pressure differential, ∆π) acts as the driving force between two liquids separated by a selectively permeable membrane. As a result, permeation of water occurs through the membrane from the lowest to the highest solute concentration solutions (i.e., feed and draw solutions respectively), while most of the solute molecules or ions are rejected [34]. As such, FO initially appeared very promising for extraction and purification of water at a low energy cost for a variety of applications, such as food concentration, wastewater concentration, water reuse and seawater desalination [34]. This sparked intense research, as demonstrated by the exponential increase of publications in recent years [35]. Several reviews have been published since 2005 discussing the interests, principles as well as the limitations and challenges for future development of the FO process. Those review more specifically discussed mass transfer limitations [34,36,37], membrane developments [36,38], fouling [39], rejection of trace organic contaminants [40], optimised draw solutions [38,41,42], energy aspects [43,44], potential applications [34,38] including wastewater treatment [35], desalination [44] and hybridisation of FO with other processes [45]. However, so far, none of this review has been dedicated to the potential application of forward osmosis in the context of combining desalination and water reuse.

Although FO on its own can be considered as a low energy process, the applications for which it can be used as a stand-alone process are limited. In fact, pure water extracted from the feed solution is only transferred to a (draw) solution with a higher osmotic potential, and as such, is rarely usable as is. A second process in which water is extracted from this solution is thus required, and this is generally the energy intensive step. FO has been initially considered using artificial draw solutions with very high osmotic pressure [34], but the need to regenerate the artificial draw solution negatively may affect the financial viability of many applications (energy costs of draw reconcentration systems such as RO or membrane distillation [44,46,47] and costs of draw replenishment due to draw solution leakages [42]). In addition, due to the closed-loop configuration and imperfect rejection of membranes, contaminants accumulation may occur [48]. Alternatively, new applications have been developed more recently to avoid the draw re-concentration step by combining existing streams and hybridising FO with other processes in once-through systems, not in closed loop [45].

### 2.2. The FO-RO Hybrid Process

**Figure 3:**
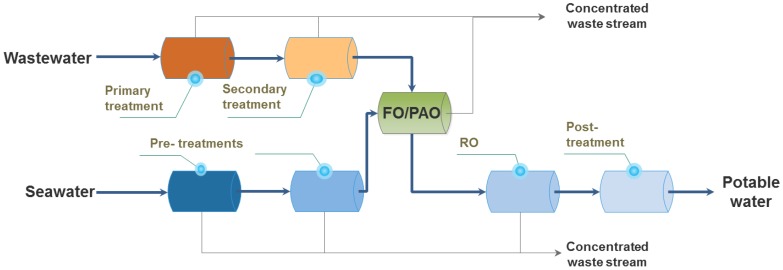
Proposed integration of FO in desalination process as FO-RO hybrid (adapted from [55]).

The interest in combining wastewater and seawater streams was only recently sparked by the new developments in FO. Combining water reuse and SWRO has been referred to as the FO-RO hybrid (Figure 3 and [49,50]) or osmotic dilution [51]. The FO-RO hybrid discussed in this study has to be distinguished from other closed-loop FO concepts used for desalination, as the FO-RO hybrid is a once-through system that does not require recovery of a highly concentrated draw solution [52,53,54]. In the FO-RO hybrid, water is transferred from an impaired water source (a low salinity feed solution, e.g., secondary effluent) to seawater (used as draw solution) by the osmotic gradient in the FO step.

In the first study focusing on this FO-RO hybrid (and the use of secondary or tertiary treated effluent for seawater dilution), the authors demonstrated that the concept could lead to four major benefits over stand-alone seawater RO desalination [55]:

- lower energy use (due to lower operating pressure) for SWRO desalination thanks to the osmotic dilution,
- beneficial reuse of wastewater, i.e., water recycling,
- Multi-barrier protection (two successive dense membrane processes, i.e., FO and RO) to increase consumer confidence in water recycling,
- Reduction in RO membrane fouling due to dilution of the pollutant load and lower operating pressure.

### 2.3. Other FO-RO Hybrid Configurations

Bamaga et al. proposed to combine the FO-RO hybrid described in Figure 3 with a second FO stage implemented as an RO post-treatment [49]. In this configuration, the additional FO stage is used to dilute the RO brine with the concentrated impaired water from the first FO step to (1) further concentrate the wastewater stream and so facilitate its post-treatment (for example via digestion) and (2) dilute the RO brine before disposal to limit its environmental impact. Although the additional FO presents some potential environmental benefits, the economic and technical feasibility is questionable due to the low permeation fluxes observed, especially in the second FO. Ultimately, recommendations to focus on the first FO stage and optimisation of module design were given [49].

FO has been demonstrated to be a robust and simple process allowing to treat difficult streams such as anaerobic digester centrate or sludge [56,57], and as such could also be well adapted to treat difficult wastewaters, mainly due to its low fouling propensity. Thus, instead of using secondary treated wastewater (Figure 3), new concepts have emerged to consider the implementation of FO upstream in the wastewater treatment scheme, i.e., on primary treated wastewater or even the direct implementation on raw sewage. It is expected that thanks to the avoidance of some purification steps, significant cost reduction could be obtained.

One example is the concept of osmotic membrane bioreactor (OMBR) [58] where FO is implemented within the secondary (biological) treatment. The OMBR has been developed by analogy with membrane bioreactors (MBRs), where biological degradation and clarification were operated in a single step. However, instead of using a porous ultrafiltration or microfiltration membrane as for MBR, a dense FO membrane is submerged in the bioreactor of the OMBR. As such, higher rejections of contaminants were observed than for MBRs, yet at lower fouling propensity [59] and thus OMBRs can produce the high water quality which is crucial in the context of potable water reuse. One major limitation in OMBR operation remains the salt accumulation in the OMBR tank, resulting from the high rejection of dissolved solids by the FO membrane and the reverse solute diffusion occurring in the FO process [60]. This salinity build up can only be mitigated by the development of more selective membranes, or by decreasing the sludge retention time. Another proposed solution was the addition of ultrafiltration or microfiltration system to OMBR to create salt bleeding, but this process is more complex to operate since two sets of well-balanced membrane systems are needed [61,62].

**Figure 4:**
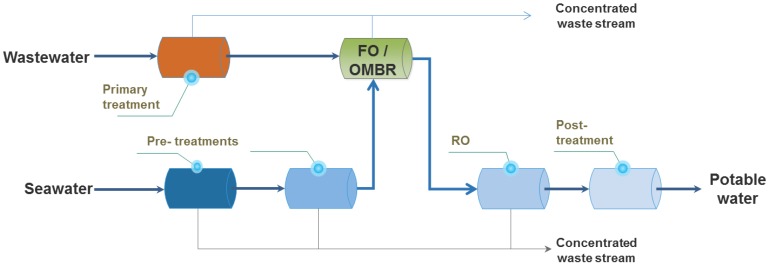
Schematic of use of FO as a standalone process or together with a secondary (biological) treatment (OMBR).

As the first experiences with the OMBR operating in a secondary biological treatment are positive, with little fouling observed, it is of course interesting to envision FO treatment further upstream, for example on the raw wastewater (or sewage) after primary treatment, as stated above (and shown in Figure 4). The interest of the water treatment community in the scheme in Figure 4 is high, as FO offers a double advantage here: not only can high quality water be recovered, in addition the concentrated sewage stream can be more easily converted to energy via digestion (due to the higher COD concentration) [63]. Initial experiments using FO on primary treated (screened) wastewater demonstrated that the accumulated fouling layer was loose and easily reversible [63], and thus fouling can indeed be controlled. Further validations are of course required, especially with regards to clogging issues in the feed channels, and also in terms of long term behavior—but implementing FO directly after primary treatment in the future could allow for significant savings in wastewater (and moreover water reuse) treatment costs.

The use of FO, after primary treatment, for sewer mining was also envisioned [35]. Sewer mining consists of a decentralised water recycling system where water recovery from sewers is envisioned for local reuse [64]. For sewer mining to be fully decentralised, FO should be combined with on-site reconcentration of draw solutions such as RO, electrodialysis or membrane distillation [65]. The latter renders the system more complex to operate and less economically favourable. Combining sewer mining with seawater desalination (i.e., using seawater as a draw) in decentralised regions avoids the need for a reconcentration step, but does require the transport of seawater to the decentralised sewer—again lowering economics. Still, similar system (using FO with seawater used as draw) has been proposed to treat urban water run-off in a coastal region, whereby FO was implemented with a seawater draw solution in decentralised concentration ponds [66]; then the diluted seawater was desalinated in the nearby SWRO plant.

### 2.4. FO Integration in Existing RO Desalination Schemes

**Figure 5:**
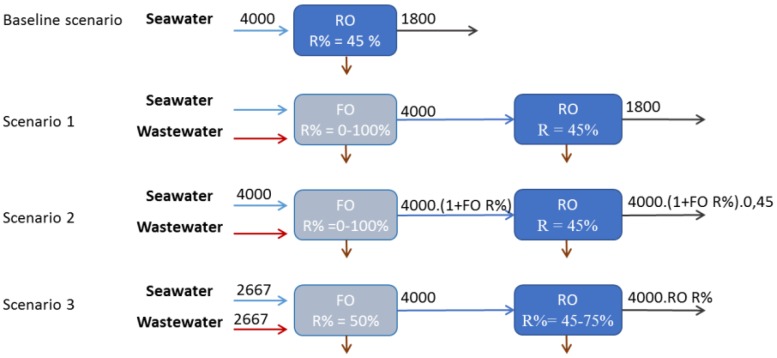
Examples of block flow diagrams of baseline (existing RO) and FO-RO with recovery (R%) for each scenario and impact on the produced water depending on FO recovery. All flow values are in m^3^·h^−1^, initial assumption of existing RO desalination plant with water production of 1800 m^3^·h^−1^ and RO recovery of 45%.

Based on the process described in Figure 3, FO can be considered as an additional treatment in an already existing seawater RO desalination scheme, resulting in salinity decrease of the seawater fed to the RO. Consequently, the lower RO feed salinity can be used (1) to produce water at lower energy cost, (2) to augment the overall water production or (3) a combination of thereof. In term of process configurations, the above options were translated in three different scenarios and compared to the baseline of stand-alone SWRO in [67] and Figure 5. When energy reduction is the primary objective, scenario 1 is preferred (with an as high as possible dilution of the seawater by wastewater) due to the significant decrease of RO operating pressure [67].

However, water reuse is exactly of high interest in those cases where water scarcity is present, to allow for water augmentation. As such, FO-RO hybrids could also be of interest when a community who already relies on seawater desalination is in need for augmenting its water supply. In that regards, scenarios 2 or 3 from Figure 5 may be preferred. FO-RO hybrid economic interest for 50% water augmentation was considered in [68] as a possible alternative to additional seawater desalination or direct potable water reuse.

As a result, the rationale for investing in FO-RO hybrid could be multiple and the economics highly variable depending on the objectives, the local context and the availability of existing water streams. Still, it is clear that a better assessment of current and future FO economics is of primary interest to support future investments in the technology.

### 2.5. FO Economics: Need for Higher Permeation Flux

Initial attempts demonstrated that FO-RO hybrids in literature can have positive economics compared to stand-alone SWRO, due to energy savings (osmotic dilution) and maintenance savings resulting from lower fouling tendency estimated from laboratory or pilot scale testing [42,55,69]. However, those initial studies did not account for some drawbacks and challenges of the hybrid that need to be considered as well [67] in order to have a fair assessment of FO-RO hybrids compared to SWRO:

- Implementation of FO will require investment costs
- Energy consumption in RO is getting close to the thermodynamic limit and additional energy savings may become marginal [70].
- The FO-RO hybrid also has to demonstrate advantages in comparison with two independent and established water treatment streams (i.e., water reuse and/or desalination) or simple mixing of these streams before treatment [71].

Several attempts were made to make a clear economic assessment of FO-RO hybrid systems. Cath et al. completed a first economic evaluation of their proposed hybrid FO-RO system by comparing the implementation of an FO unit to an expansion of SWRO capacity, to increase seawater desalination plant capacity. Their estimations showed 0.43US $·m^−3^ cost savings of the FO-RO hybrid compared to stand-alone RO. However, this estimation was based on the assumption of a high energy cost, and investment costs of FO only related to membrane costs [50]. Another study revealed that FO can be a viable technology thanks to significant energy decrease from 2.5 to 4 kWh·m^−3^ for RO seawater desalination down to 1.5 kWh·m^−3^ when using FO-RO hybrid [72]. However, this requires a dilution of seawater by a factor of 2.5, the process relies then mostly on water reuse.

It is clear that the key to improving FO process economics is in the increase of FO fluxes [38,42,67]. A recent and complete study established that FO-RO hybrid systems (operated in once-through, not in closed loop), will only become economically sustainable if lower membrane costs (30 US $·m^−2^) and/or higher fluxes (≥15 L·m^−2^·h^−1^) than for existing commercial membranes can be obtained [51]. Another recent study confirmed that the current state of development of commercial FO membrane modules is insufficient for sustainable FO-RO hybrid economics due to the high capital investment cost (CAPEX—which is related to low permeation flux, low packing density, and high membrane costs) [67]. A threshold flux value of 30 L·m^−2^·h^−1^ was proposed as minimum average permeation flux to guarantee FO economic sustainability. 

A very recent study, using levelised cost indicator, demonstrated that FO-RO hybrid could be a favourable alternative for 50% water augmentation in comparison with extension of SWRO or implementation of DPR [68]. This study also showed that the economic viability of the FO-RO hybrid was highly dependent from the extent of wastewater treatment required and the permeation flux in FO.

Despite a significant body of research on the development of tailor-made membranes, until very recently, only few commercial membranes were available. A new generation of membranes and modules is now emerging on the market, but their economics and performance still need careful assessment (See Section 3). As an alternative to FO, the concept of pressure assisted osmosis (PAO) has arisen recently, and appears promising to overcome the current flux limitations of FO. Opportunities and challenges of novel membranes, novel modules and the use of PAO operation, to improve permeation flux in osmotic processes, are critically and systematically discussed in the following sections.

## 3. Recent Development to Improve Flux in FO

### 3.1. Membrane Development

Permeation flux in FO is largely dependent on membrane characteristics. FO membranes are usually asymmetric polymeric membranes. The parameters used to characterize these membranes are typically the pure water and salt permeability of the rejection layer (factors A and B, respectively), and the structural parameter of the support layer (S). The ideal FO membrane features a high A value (high water flux), low B (low salt passage), low S (to limit internal concentration polarisation, ICP) and sufficient mechanical strength to support industrial operation at moderate pressure [34]. Since the introduction in the 1990s of the first FO commercial membrane by HTI, a tremendous amount of work has been performed to optimise FO membrane [38]. Two main strategies have been followed in this respect: (1) developing dedicated membranes for FO or (2) adapting existing NF/RO membranes.

The first strategy was by far the most studied and recent reviews reported on numerous membrane developments that have been published since 2005 on both hollow fibre and flat sheet configurations [36,38,43]. Among them, new approaches have been used to develop thin-film composite (TFC) membranes which consist of a selective polyamide layer formed by interfacial polymerisation on top of a polysulfone porous substrate [73], similar to NF/RO membranes. The TFC membranes offer more flexibility than cellulose triacetate (CTA) membranes in choosing active and support layer, and as such TFC membranes with higher permeability and reduced ICP were synthesized, allowing for higher water fluxes [74]. The concept of TFC membranes has been extended to the synthesis of double-skinned layer FO membranes [73], leading to lower ICP and fouling. Another approach for TFC membrane improvement was the development of hydrophilic support layers leading to lower ICP and subsequent higher water flux, but with the drawback lower salt rejection [73]. The use of nanofibres as membrane support layer to limit ICP is also a new way to improve TFC membranes [75]. Recent work also mentioned the layer-by-layer approach (LbL) that allows formulating tailor-made membranes [76,77,78,79]. In addition, several publications referred to next generations of biomimetic FO membranes using Aquaporin Z [80,81], carbon nanotubes (CNT) [82,83] or graphene [84].

The second strategy to novel FO membranes consists in adapting existing RO membranes. Such membranes exhibit high permeabilities and high salt rejection, but have the drawback of a thick, porous hydrophobic support layer [85] which is inadequate for FO due to the severe ICP occurring [86,87]. Thus, membrane support layers were modified by removing the backing support layer [87], or by improving wettability using polydopamine coating [88]. Water flux was increased by up to 10 times in comparison with the parent RO membranes at high osmotic driving forces. The use of conventional NF membranes in FO applications was also proposed in 2007 [89]. Then, a number of studies [78,79,89,90,91,92,93] considered the development of FO membranes with more porous active layers, similar to those found in NF membranes, to increase water permeability. However, the water flux obtained from the modified NF-FO membrane was not deemed high enough, also mainly due to ICP. Additionally, the reversible salt diffusion (RSD) values reported for NF-like FO membranes are generally high or require the use of divalent salts as draw solutions [78,79,92,93].

Overall, findings from academic research have been translated into the development and (pre-) commercialisation of several FO membranes—described hereafter in Table 1. It has to be noticed that HTI, which has been the main leader in FO membrane development and main provider to academic research, is no longer capable to supply membranes [94]. Other membrane suppliers nowadays offer membrane samples but information in the literature remains limited due to their more recent development.

Based on information available (Table 1), it can be noticed that most of the development has focussed on TFC flat sheet membranes; Hollow fiber (HF) membranes are still in the development phase, and have not been commercialised to a high extent yet. Novel flat sheet membranes which have incorporated the TFC approach, and several new biomimetic membranes (CNT and aquaporin) are now commercially available [95]. As a result, several companies (Porifera, Woongjin Chemicals, CSM-Toray, Oasys Water) claim water permeation fluxes of around 30 L·m^−2^·h^−1^ when using 1 M (NaCl, KCl) draw solutions, with reverse salt diffusion being below 1 g·L^−1^. Such performances represent a significant improvement in comparison with the HTI CTA membrane, which still acts as a reference. It is clear that these novel membranes will surely help to further develop FO applications.

### 3.2. Module Development

**Figure 6:**
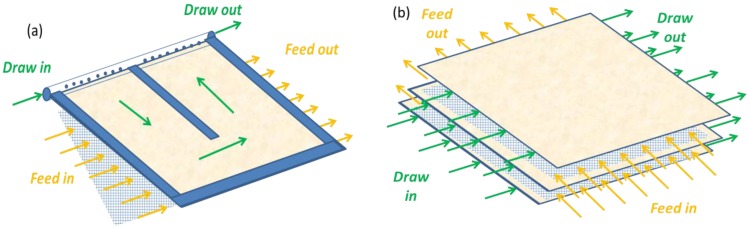
Illustrations of flat-sheet FO membranes arranged in (**a**) spiral wound and (**b**) plate and frame modules design.

Among the challenges to overcome in FO, module design is certainly of a high importance. An ideal FO module is expected to demonstrate an appropriate trade-off between (1) a maximised surface area (i.e., high packing density) and (2) a minimised pressure drop, while (3) allowing for limited ECP and particle deposition [35,63]. In the early stage of FO and PRO research, FO modules were mainly adapted from RO configurations, but these spiral-wound modules proved to have limited efficiency as a result of imperfect hydraulics on the permeate (draw solution) side [34,104,105]. Indeed, FO modules differ from classical RO ones as fluids (feed and draw solutions) have to circulate on both sides of the membrane. As such, FO modules require four ports (feed and draw inlets and outlets) and optimised hydraulics on the feed and the draw side. Some examples of flat sheet FO module arrangement are described in Figure 6.

The advantages and disadvantages of three modules available for FO, i.e., plate and frame, hollow fibre and spiral-wound module configurations, have already been extensively described elsewhere [35,63]. In practice, the following configurations have been commercially developed by FO membrane suppliers:

- Plate and frame modules are developed by Porifera under the commercial name of PFO elements. Porifera PFO elements are claimed to offer relative high packing density (similar to RO modules), low pressure drop and filtration surface from 1 up to 7 m^2^ per module [102]. Up onto now, however, the performance of these modules has not been systematically reported in literature.
- Hollow fiber modules were developed by Toyobo, as adapted from their 8 inch SWRO modules. These modules featuring high packing density have been tested for PRO [104], and the authors provided proof for resistance to operation at high hydraulic pressure (25 bar). However, the authors did mention the need for optimised design through adapted flow patterns for both feed and draw sides.
- Spiral wound modules were developed by HTI using the CTA FO membrane. The modules were developed in a range of module sizes, varying from 2.5 to 8 inches, and for a variety of feed spacers (fine (FS), medium (MS) and corrugated (CS) spacer) to allow for operation with different types of feed waters. Most FO studies on pilot scale were performed using the HTI CTA modules (see Table 2).

Other companies such as Oasys Water and Modern Water also provide full scale solutions and thus large FO modules (Toray Inc. is also supplying 8 inches SW modules) but their module configurations are not explicitly described in open literature yet [106].

One of the main gaps in knowledge of FO modules is the impact of hydraulic pressure on the performance. The main reason why this has not been systematically investigated is that FO is considered as an osmotic driven process. However, one cannot ignore pressure drop along modules inherent to any membrane processes practical implementation. In fact, mass transfers are usually optimised by the implementation of spacers that create turbulences and consequently limit particle deposition and concentration polarisation, but at the cost of additional pressure drop [112,113]. Only one study from Kim et al. in 2011 ([109], Table 2) mentioned the impact of feed and draw CFV on pressure drop and pressure build up in spiral wound FO modules. A minimum amount of hydraulic pressure (0.12 and 0.28 bar on feed and draw sides respectively) needed to be applied to allow water to flow through the module even at the lowest flowrates. It was clearly demonstrated that CFV and channel pressure drop were closely connected in both channels (feed and draw), and were highly dependent on the spacer type used. Additionally, it was observed that applying pressure on the feed side lead to a narrowing of the draw channel and consequently, to a pressurisation of the draw side. Similar observations were also described in PRO configuration where pressurisation of the draw channel led to narrowing of the feed channel when diamond shape spacer was used to support the membrane [114]. In fact, as for other membrane processes, spacer design is of crucial importance. Among the modules proposed by HTI in former studies, even if not always specified, at least two types of draw spacers have been tested, i.e., permeate carrier [109,110] and RO feed spacer [107]. It is also generally observed that modules are operated at very low CFV on the draw side (Table 2), maybe thanks to the low fouling behaviour draw solution but also possibly limited by the important pressure drop occurring when permeate carrier are used [110].

Not many studies have tried to use computational fluid dynamics (CFD) for FO module design yet. In addition, among the few studies reporting CFD approaches in FO, none of them considered the impact of pressure in the different spacer-filled channels. Most of the approaches were dedicated to the demonstration of models capable of simulating FO systems [115,116] or to demonstrate current mass transfer limitations, the need for improvement of membrane separation properties and the study of spacer designs to limit ECP [117,118].

More work is thus required to better understand how CFV, spacer type and module configuration are connected to pressure drop and hydraulic pressure in the spacer-filled channels, to determine the optimum configuration for FO up-scaling. CFD modelling could help in further understanding mass transfer limitations in FO modules and to propose optimised designs.

### 3.3. The Concept of Pressure Assisted Osmosis (PAO)

**Figure 7:**
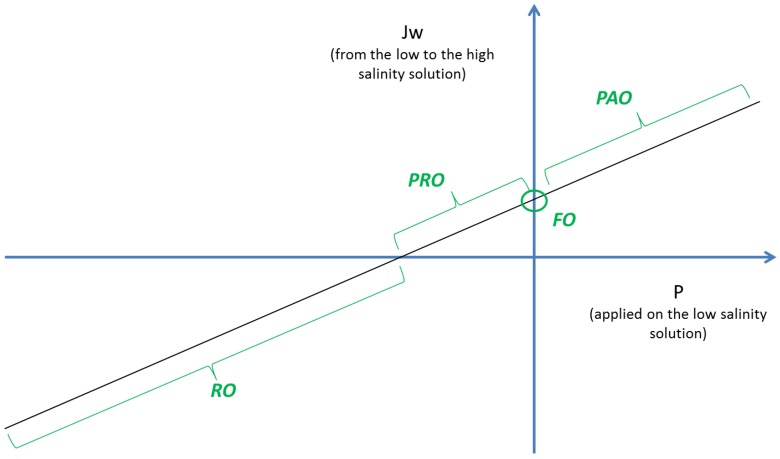
Illustration of water fluxes obtained (J_w_) in osmotic processes as a function of hydraulic pressure applied (P) on the low salinity solution, and the potential of PAO to provide high fluxes.

The concept of pressure assisted osmosis (PAO) [119], relies on the application of moderate pressure on the feed side of a FO system to enhance water permeation through the membrane (Figure 7). As such, by a synergistic effect of hydraulic and osmotic pressure, PAO can improve FO fluxes and thus FO process economics due to lower membrane surface requirements.

The impact of hydraulic pressure on the feed of FO systems was only studied recently. The first study mentioning hydraulic pressure on the feed side was presented as a conference paper in 2011 and already discussed the interest of pulsations and moderate hydraulic pressure to improve permeation flux [120]. The same year, another study also showed that hydraulic pressure, even if very moderate, is needed in FO systems to allow water cross-flow within the feed and draw channels of an FO module [109]. The effect of transmembrane pressure in FO was further evaluated, assuming that FO industrial applications require pressurisation for water circulation within spiral wound modules [97]. However, given the low applied pressure (up to 3.4 bar) in comparison with the osmotic pressure driving force (45 bar),no clear impact on flux was observed for the three membranes tested.

The implementation of hydraulic pressure in FO as a concept only appeared in 2012 and was initially named ‘’pressure assisted forward osmosis’’ [121], also later on called ‘’assisted forward osmosis’’ [122] and ‘’pressure assisted osmosis’’ (PAO) [123,124]. Initial research using HTI CTA membranes confirmed flux improvement as a result of PAO operation when compared to FO [121,122]. On the one hand, it was observed by some that the water flux increment remained lower than expected by the additional driving force, attesting for enhanced ICP partly mitigating the beneficial use of hydraulic pressure, and thus indicating that PAO might not be beneficial [125]. On the other hand, evidence of membrane deformation occurring due to pressurisation of the membrane over draw channel spacers was also observed. The membrane stretching over spacers strands led to increased membrane permeability and consequently significant improvement of the water flux was observed [122]. Comparative investigations of PAO in continuous and discontinuous mode also confirmed that water flux increases with hydraulic pressure [123]. Interestingly, and as a result of more intense ICP in PAO operation, RSD decreased, tackling a second limitation of current FO operation, and thus rekindling the interest in PAO [124]. 

More recent work compared the performance of CTA membranes in PAO mode to that of commercial TFC membranes. In addition to allow for higher flux in FO process, the TFC membranes were also more responsive to hydraulic pressure applied in the PAO process, and thus showed clear flux enhancement (up to 25 L·m^−2^·h^−1^) at moderate hydraulic pressure [126]. In addition to providing extra driving force for permeation flux, hydraulic pressure was also observed to limit RSD and increase the water permeability due to membrane deformation when TFC membranes were used. Therefore, PAO constitutes a promising alternative to tackle the permeability-selectivity trade-off of FO.

## 4. Challenges Associated with FO Flux Improvement

### 4.1. Fouling and Cleaning

The behaviour of individual or combined model foulants (humic acids, alginate, proteins, silicates, calcium) under different operating FO conditions has been extensively described in the literature [60,69,127,128,129,130,131,132,133,134,135,136,137,138] and summarised in a recent extended review [39]. In these studies, it is generally observed that fouling in FO remains moderate and easily reversible. The only recommendation, to avoid irreversible fouling in the support layer when wastewater is used as feed [139] was to operate the FO membranes with the membrane active layer facing the feed solution (AL-FS) [135,140]. Up to now, most FO fouling studies were performed in FO operation without applied hydraulic pressure on the feed or draw, and mostly using the benchmark CTA HTI FO membrane, which demonstrates a relatively low permeation flux [60,69,128,129,130,131,132,133,134,135]. It is clear that increased fluxes (either due to PAO operation or by using high permeability membranes) will impact the fouling behaviour. In addition, in PAO operation there is an applied pressure (in contrast to FO), and a clear research question remains on the respective impact of flux and pressure on the fouling behaviour.

The impact of operating flux on fouling behaviour has been well studied for pressure driven membrane processes [141]. The concept of critical flux [142] has been widely used when describing the impact of flux on fouling for membrane bioreactors [143,144], and also for other hydraulic pressure driven membrane processes such as RO [145,146]. It has been demonstrated that high water permeation (above the critical flux) led to enhanced fouling; thus, operating below the defined critical flux is preferred for sustainable long-term filtration. The evidence of critical flux was first revealed for FO in first studies using HTI CTA membranes, with the support layer facing the foulant-feed solution and under elevated osmotic driving force [140,147]. Further evidence of critical flux was demonstrated in FO studies, when using the conditions that can be expected in the FO-RO hybrid system, namely operation in AL-FS mode at moderate osmotic pressure differences [135,148]. Those studies demonstrated that the low fouling behaviour often mentioned for FO is mainly due to the operation at low permeation fluxes. At higher initial fluxes, the fouling cake was more compacted on the membrane surface and consequently significant flux decline was observed over time.

Only recently, more studies have been published that consider the impact of moderate hydraulic pressure on fouling behaviour. Typically, higher fouling propensity and lower reversibility of combined organic–colloidal fouling (alginate and silica) was reported when hydraulic pressure was applied at relatively high hydraulic pressures (7–19 bar) [149]. Two recent studies [150,151] confirmed the hypothesis raised that PAO fouling was a consequence of both hydraulic and osmotic driving forces (i.e., combination of fouling cake compaction and RSD by analogy with RO and FO fouling mechanisms respectively [152]). Typically, in PAO operation, even at similar flux compared to FO operation, a thinner but more compact fouling layer than in FO is observed, leading to more flux decrease [150]. The flux decrease is higher than in FO, but still more reversible than in RO [151].

Tackling fouling is a key aspect in membrane processes and is usually achieved via a combination of fouling mitigation (i.e., membrane and module development and/or optimisation of hydrodynamic conditions) and adapted cleaning strategies [153]. FO studies dedicated to fouling mitigation via membrane surface modification and cleaning are discussed successively here.

TFC membranes developed for FO have proven to initially enhance water permeation, although their much rougher surface generally results in more fouling [154,155] as already demonstrated for NF/RO TFC membranes [156,157]. Some membrane developments have recently been dedicated to fouling mitigation such as double skinned membranes [77,158], membrane surface modification approaches using amine enriched, polyethylene-glycol enriched [77,158,159] and silver-titanium nanoparticles [160]. Some promising results have been observed, but studies remain scarce and limited to lab-scale and home-made membranes. However, it has been observed elsewhere that membrane surface properties had ultimately a low impact on fouling behaviour since it was limited to the early stage of the foulant deposition [148].

**Figure 8:**
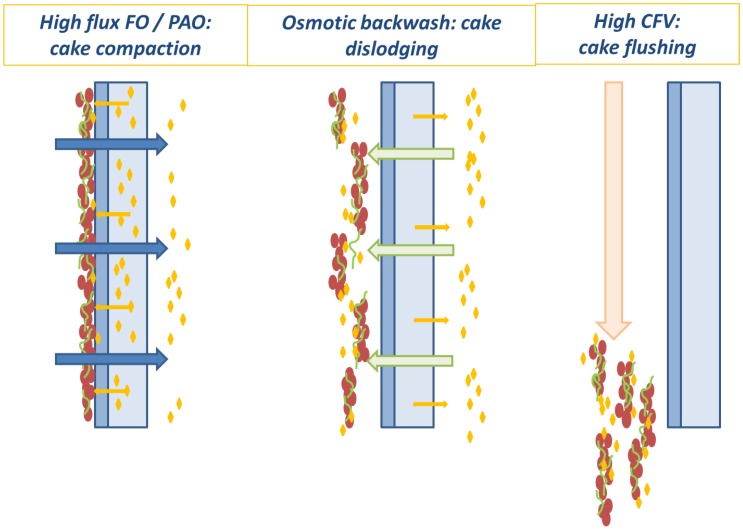
High flux FO and PAO fouling and cleaning (osmotic backwash and high cross-flow velocity flushing (adapted from [150]).

Since in FO operation at low fluxes, relatively little fouling has been observed so far, cleaning strategies in FO have mostly been limited to applying simple physical methods to improve turbulence (i.e., high CFV, use of spacers or pulsed flow [128]). Chemical cleaning and air scouring also provided positive results, but were mostly not necessary as physical cleaning proved to be sufficient [72,161]. Similarly to hydraulic backwashing used for porous membranes, osmotic backwashing has been tested for osmotic processes. For FO, the exact impact of osmotic backwashing on fouling control is unclear: some studies mention a significant recovery of initial flux after cleaning of the fouled membrane [56,162,163], while other work only observed a very low impact on the fouling removal [161,164]. Following on a former study on RO [165], a recent publication therefore proposed an optimised sequence for FO/PAO cleaning, which consists of osmotic backwashing to detach the foulant cake from the membrane surface and high CFV operation to flush the feed channel with fresh water to remove the foulants that were dislodged from the surface. This method proved to be efficient, even at high FO permeation flux and in PAO operation [148,150]. A more detailed insight in fouling and cleaning mechanisms is starting to emerge (Figure 8), which shows that even high flux membranes operated in PAO mode can be cleaned without the need for chemicals.

### 4.2. Rejection of Trace Organic Contaminants

To ensure water safety in FO-RO hybrids, TrOCs is of course of concern. TrOCs include endocrine-disrupting chemicals, pharmaceutically active compounds, pesticides, and disinfection by-products. They are present in impaired water in ng/L to µg/L levels [166,167], and could represent a human and environmental threat, even at low concentrations [48]. Recently, extended research was performed to evaluate FO as a barrier against TrOC, especially in association with RO [108]. A recent review summarised recent studies dedicated to the fate of TrOC in the FO process [40]. Among the studies cited, it was observed that the FO process may provide a robust barrier for most TrOCs, but for some TrOCs, only limited rejection was found. In addition, most of the FO studies on TrOC were carried out using the commercial HTI CTA membrane, which demonstrates relative low permeation fluxes (which could impact the low TrOC rejection).

**Figure 9:**
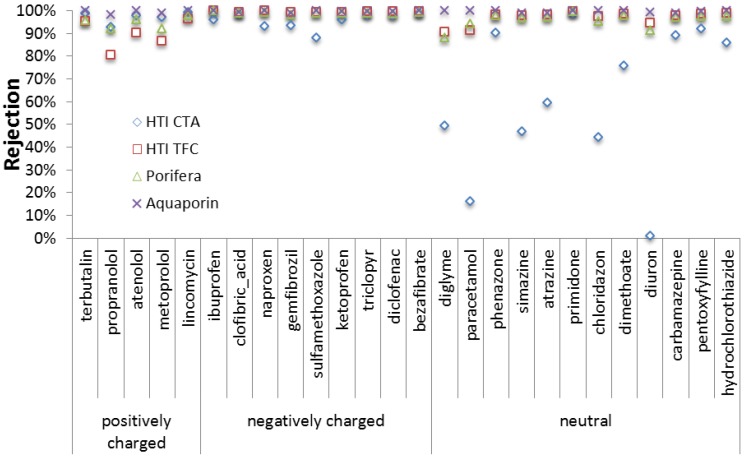
Rejection of TrOC in FO operation with four commercially available membranes (adapted from [126].

Of the novel membranes, biomimetic membranes incorporating Aquaporins have demonstrated higher rejections of small neutral organic pollutants at similar permeation flux compared to the HTI CTA [100]. The commercial TFC membrane developed by Oasys Water has also been recently evaluated with regards to TrOC rejection [168] and demonstrated higher rejections of neutral TrOC compared to the HTI CTA, which was attributed to a higher active layer structural factor and a more negative charge. Another recent study compared several membranes and confirmed higher rejections of TFC membranes compared to the HTI membrane (Figure 9), especially for the smaller neutral TrOCs (>80% rejection for all TrOCs studied for HTI TFC, >90% for Porifera, >98% for Aquaporin). The increased rejections compared to the HTI CTA were mainly due to increased steric hindrance (and thus a smaller active layer pore size). However, it was clearly shown that rejection of TrOCs dropped sharply when the membranes were operated in PAO, most likely as a result of a combination of membrane deformation under pressure, more ECP and less RSD. As such, in PAO-RO hybrids, attention has to be paid to FO membrane mechanical resistance when it comes to TrOC rejection (while deformation is interesting in terms of flux in PAO).

## 5. Concluding Remarks

FO-RO hybrid processes offer a promising solution not only to lower desalination energy needs, but also to increase water reuse efficiency by combining seawater desalination and water reuse. Interestingly, due to the lower fouling propensity compared to pressure driven membrane system, FO has the potential to treat feed water of various qualities (potentially even including raw sewage), allowing to lower wastewater treatment costs. FO-RO schemes do require further validation but also radical shift in current consideration of water supply. Societal (public perception of water reuse) and water management (proximity of wastewater and desalination plants) challenges clearly need to be overcome.

This review clearly emphasized the need for flux increase to allow for more favourable FO economics and discussed the required technical development (i.e., novel membranes, PAO mode). However, flux improvement is of course also associated with drawbacks, such as increased fouling, lower rejection of TrOCs in PAO operation, and the limits of membrane mechanical resistance.

At this stage, it is, therefore, questionable if the FO/PAO-RO hybrid process will allow sustainable and long-term operation at high flux. Additional studies are required to support successful implementation of FO-RO hybrids in the industry:

- Up-scaling: most of the studies in literature have been conducted using small flat-sheet coupons. More pilot scale and full scale tests are needed to assess up-scaling challenges in term of mass transfer limitations on module scale, the effects of spacer design on pressure drop, effects of fouling and the feasibility of cleaning strategies.
- Improved economic assessment: The economic models used for FO should be updated by incorporation of fouling models that are better able to simulate practical implementation of FO/PAO-RO hybrids. In addition, a better integration of cost savings from the water recycling scheme may be considered as any treatment step avoided in the water recycling scheme as a result of combination with desalination will help to support FO/PAO-RO hybrids economic credentials.

## Figures and Tables

**Table 1:** Development and performance of commercial FO membranes (performance as seen in the literature with deionised water (DI) as feed and active layer facing feed solution (AL-FS) used as membrane orientation (data compiled in July 2015)).

Company	Type	Commercial Name	Status	FO Performance			Ref.
				Draw	J_w_	J_s_/J_w_	
					L·m^−2^·h^−1^	g·L^−1^	
HTI	flat-sheet	CTA-NW	commercial	2M NaCl	8.5	0.1	[96]
HTI	flat-sheet	CTA-ES	commercial	1M NaCl	10.1	0.5	[97]
HTI	flat-sheet	TFC	commercial	1M NaCl	10	0.8	[97]
Oasys	flat-sheet	TFC	pre-commercial	1M NaCl	30	0.7	[97]
Woongjin Chemicals	flat-sheet	TFC-1	development	1M KCl	16	1.3	[98]
Woongjin Chemicals	flat-sheet	TFC-2	development	1M KCl	27.9	0.4	[99]
Aquaporin	flat-sheet	AqP	pre-commercial	1M NaCl	9.5		[100]
CSM-Toray	flat-sheet	FO membrane	commercial	1M NaCl	35.0	<0.5	[101]
Porifera	flat-sheet	PFO elements	commercial	1M NaCl	33.0	0.2–0.6	[102]
Samsung	hollow fiber	HFFO lumens	development	1M KCl	9.3	0.6	[103]
Toyobo	hollow fiber	–	commercial	–	–	–	[95]

**Table 2:** Reported module configurations and operating parameters for HTI modules (CFV: cross-flow velocity).

Module	Feed Spacer	Draw Spacer	Filtration Surface (m^2^)	CFV Feed (cm·s^−1^)	P Feed (bar)	CFV Draw (cm·s^−1^)	P Draw (bar)	Ref.
Prototype	RO feed spacer	RO feed spacer	0.94	0.1	n.r. ^a^	0.1	2	[107]
4040	2.5mm RO feed spacer	n.r.^a^	1.58	5	n.r. ^a^	1.5	n.r. ^a^	[108]
4040-MS	1.14mm RO feed spacer	Permeate carrier	3.2	16	1.22	4.3	1	[109]
8040-MS	1.14mm RO feed spacer	Permeate carrier	11.2	62	n.r. ^a^	0.4	2	[110]
8040-CS	2.5mm RO feed spacer	Permeate carrier	9	30	<1	0.4	<0.7	[110]
4040-MS	1.14mm RO feed spacer	n.r. ^a^	3.3	15	0.7–1.1	10.0	0.5	[111]

^a^: Not reported.
